# Correlating maternal iodine status with neonatal thyroid function in two hospital populations in Ghana: a multicenter cross-sectional pilot study

**DOI:** 10.1186/s12887-020-1932-6

**Published:** 2020-01-21

**Authors:** Selorm A. Dei-Tutu, Adoma Manful, Douglas C. Heimburger, Hawa Malechi, Daniel J. Moore, Samuel A. Oppong, William E. Russell, Muktar H. Aliyu

**Affiliations:** 1 Ian Burr Division of Pediatric Endocrinology and Diabetes, Vanderbilt University School of Medicine, Nashville, TN USA; 2 Baylor College of Medicine, Section of Pediatric Diabetes and Endocrinology, Houston, TX USA; 3 Vanderbilt University School of Medicine, Nashville, TN USA; 4 Vanderbilt Institute for Global Health, Nashville, TN USA; 5 Department of Obstetrics and Gynecology, Tamale Teaching Hospital, Tamale, Ghana; 6 Department of Obstetrics and Gynecology, Korle-Bu Teaching Hospital, Accra, Ghana

**Keywords:** Iodine deficiency, Congenital hypothyroidism, Ghana

## Abstract

**Background:**

Congenital hypothyroidism is a common, yet easily treatable cause of poor growth and intellectual disability. Newborn screening programs play an important role in the early detection and treatment of congenital hypothyroidism. However, an estimated 71% of children are born in countries such as Ghana, which does not have a screening program. Iodine deficiency, a common cause of congenital hypothyroidism, is present in the Ghanaian population. Mild to moderate maternal iodine deficiency may negatively impact cognitive function in children. A structured approach to examine the association between maternal iodine levels and infant thyroid function may have important ramifications on our understanding of congenital hypothyroidism in Ghana. We investigated the hypothesis that maternal iodine deficiency impacts infant thyroid function, using Thyroid Stimulating Hormone (TSH) as a marker of thyroid function. We also explored potential opportunities and barriers to newborn screening for congenital hypothyroidism in Ghana.

**Methods:**

This was a cross-sectional, multicenter pilot study of 250 women and their neonates recruited from post-natal clinics in Accra and Tamale, Ghana. We compared maternal urine iodine concentration and infant TSH, as well as maternal sociodemographic and nutrition information. Regression models were used to model the relationship between variables.

**Results:**

Median infant TSH was 4.7 μIU/ml (95% CI: 3.9–5.5) in Accra. In Tamale, the median infant TSH was 3.5 μIU/ml (95%CI: 3.3 to 3.6) (Δ: 1.3 μIU/ml, 95% CI: 0.5–2.1, *p* = 0.002). Median maternal urine iodine concentrations were 141.0 μg/L (95% CI: 115.7 to 166.3) and 142.5 μg/L (95% CI: 125.1 to 160.0) in Accra and Tamale, respectively (Δ: − 1.5 μIU/ml, 95% CI: − 32.2 – 29.2, *p* = 0.925). There was a weakly positive correlation between maternal urine iodine and infant TSH (rho 0.1, *p* = 0.02). Almost one-third (30%) of women in both locations had biochemical evidence of iodine deficiency. Mothers with any formal education were more likely to have higher iodine levels than their counterparts who had no formal education (coefficient 0.31, *p* = 0.006).

**Conclusions:**

Maternal iodine deficiency is prevalent in Ghana and is correlated to infant thyroid function. We recommend studies with larger sample sizes to assess the true scope of this relationship.

## Background

Across the world, congenital hypothyroidism is a common, yet easily treatable cause of poor growth and intellectual disability. Clinical features of hypothyroidism in infants include lethargy, wide fontanelles, flattened nasal bridge, macroglossia, bradycardia, protuberant abdomen with a large umbilical hernia, hypotonia, and delayed reflexes. Patients with congenital hypothyroidism can also have severe constipation, feeding problems, prolonged jaundice, poor growth, as well as profound intellectual disability [1, 2]. There is a delay in the onset of clinical signs, therefore many neonates with congenital hypothyroidism are diagnosed clinically until 3 months of age or older. However, the insufficient levels of thyroid hormones cause significant and irreversible damage to the developing brains of affected infants, in spite of this lack of early clinical signs. Hence, early and aggressive treatment, ideally initiated within the first 2 weeks of life, is important for the protection of cognitive function [1–3].

Many countries have newborn screening programs (NBS) to facilitate early detection and treatment of congenital hypothyroidism. Laboratory screening approaches include testing with one of the following: 1) primary thyroid stimulating hormone (TSH) measurement; 2) primary thyroxine (T4) measurements; or 3) combined TSH plus T4 measurements [1]. Dried blood spots on filter paper is the preferred means by which samples for newborn screens are collected. Based on current clinical recommendations a newborn who tests positive for congenital hypothyroidism on a newborn screen should have confirmatory testing and initiation of therapy with levothyroxine within 2 weeks [3]. The later the initiation of therapy, the more severe the deficit [3]. In spite of advances and expansion of newborn screening however, the most current estimates suggest that 71 % of children are born in parts of the world, such as Ghana, which do not have an established newborn screening program [1, 4]. This situation makes children in these places vulnerable to otherwise easily preventable sequelae of hypothyroidism.

Iodine is crucial for the synthesis of thyroid hormones which modulate growth, development, and metabolism of organisms [5]. Thyroid hormones are particularly crucial in the growth and development of the fetal brain. Maternal iodine deficiency is a major cause of congenital hypothyroidism [6–9]. Iodine deficiency and its resulting thyroid dysfunction also have repercussions on the health and well-being of adults, with particularly adverse effects on reproductive and perinatal health, resulting in effects such as increased infertility and perinatal mortality [10–12].

Iodine deficiency remains a significant public health concern in Ghana. In the 1990s, the Ghanaian government began large-scale salt fortification with the goal of providing at least 90% of the population with iodized salt. Data from 2008, however, showed that while the prevalence of goiter decreased, only 74% of households consumed iodized salt [13, 14]. Consumption of iodized salt has been reported to be particularly low among inhabitants of the Northern region of Ghana [15].

Essentially all ingested iodine is excreted in urine, making urine iodine concentration a sensitive, easily obtainable, and affordable means of measuring the iodine status of an individual or a population. Though an individual’s urine iodine concentration may vary daily, these differences even out on a population level, and urine iodine concentration remains the most widely accepted means of testing iodine levels. Urine iodine concentration less than 150 μg/L is considered insufficient for pregnant women, whereas urine iodine concentration less than 100 μg/L is considered insufficient for lactating women and children under the age of 2 [16, 17]. Mild to moderate maternal iodine deficiency may negatively impact cognitive capabilities of children [18, 19]. Hence, examining the association between maternal urinary iodine concentration and infant TSH levels will improve our understanding of congenital hypothyroidism in Ghana, and advance the case for universal newborn screening and for improved iodine supplementation, particularly during pregnancy. This study assessed the impact of maternal iodine insufficiency on neonatal thyroid function, using TSH NBS as a marker of thyroid function.

## Methodology

### Study design

This study was a multicenter, cross-sectional pilot study of 250 women and their 257 infants, carried out at the Korle-Bu Teaching Hospital (KBTH) in Accra and the Tamale Teaching Hospital (TTH) in Tamale, Ghana. All eligible participants for the study were approached and recruited if they presented at the 1 or 2-week postnatal clinics. Mothers and infants were excluded from enrollment if the infant was younger than 3 days or older than 30 days old. Additionally, mothers with known chronic diseases that could impact urine iodine concentrations, such as chronic kidney disease or history of intake of iodine-containing medications, were excluded.

### Definitions

A newborn screen for congenital hypothyroidism was considered positive if the infant filter paper TSH was > 17 μIU/ml in whole blood [20, 21]. Based on the World Health Organization’s (WHO) classification, maternal urine iodine concentration was further disaggregated into: severe iodine deficiency (< 20 μg/L); moderate iodine deficiency (20–49 μg/L); mild iodine deficiency (50–99 μg/L); sufficient iodine level (100–199 μg/L); above requirements (200–299 μg/L); and excessive iodine intake (> 300 μg/L). Mothers were considered iodine deficient if their urine iodine concentration was < 100 μg/L [16].

### Recruitment/procedures

In Ghana midwife-obstetrician teams oversee the first two post-natal visits, typically at 1- or 2-weeks post-partum and again at 6-weeks post-partum. At the beginning of each clinic, midwives lead a group class on a topic of their choice. Research nurses for this study, midwives who work at their respective sites, led the group class during recruitment periods. They taught clinic attendees about the potential effects of maternal iodine deficiency on children and introduced the research study during this group class. After the class, women who indicated interest in the study were further screened to determine eligibility. Mothers who came to the clinic after the class was over were also approached individually, informed about the study and screened for eligibility, if they indicated interest.

Informed consent was obtained from mothers in English or in the local languages of Ga, Twi, Dagbani, and Hausa, as needed. Urine samples were collected from mothers, transferred into small tubes and frozen within 6 h of collection. Frozen samples from each site were then batched and sent to the urine iodine lab at the end of the 8-week data collection period. Samples from both sites remained frozen throughout the shipping process.

The urine iodine concentration test was run by the Micronutrient Research Laboratory of the Department of Nutrition and Food Science of the University of Ghana. This lab is certified by the U.S. Centers for Disease Control and Prevention Ensuring the Quality of Urinary Iodine (CDC EQUIP) program for iodine laboratories, supporting numerous projects and countries. Urine iodine concentration was determined using the Sandell-Kolthoff reaction [17].

Heel-stick blood samples from infants were collected on filter paper. The filter paper cards were labelled, dried for at least 1 h, and placed in individual Ziploc® bags with desiccant. Samples in both locations were stored in a cool, dry, secure location. In Accra, the samples were sent directly to the laboratory within 6 h of collection. All samples from Tamale were stored in a laboratory within 6 h of collection, then batched and shipped to Accra at the end of the data collection period. TSH levels were determined using ELISA assays on the dried blood spots. The TSH ELISA kits were manufactured by RayBiotech, Norcross, GA. The kits were shipped to Ghana on dry ice via commercial courier service, and the assays were run by the Chemical Pathology laboratory at the Department of Medical Biochemistry, School of Basic and Allied Health Sciences.

We collected data from mothers, including demographics such as maternal age, age of infant, gestational age of infant and history of multiple gestations; nutritional information such as whether iodized salt is used in the home; and clinical information, for example, medications taken in pregnancy, complications of pregnancy and delivery, history of goiter, and delivery type [22]. Research staff reviewed maternal antenatal and delivery charts to obtain information to supplement the participant interview. Data were managed using the Research Electronic Data Capture (REDCap), an electronic data management system hosted at Vanderbilt University [23].

### Power analysis

We used the Pearson correlation coefficient method for power analysis. A sample size of 150 provides at least 90% power to detect a clinically significant correlation between maternal urine iodine levels and infant TSH, with a correlation coefficient of 0.6 and a 2-sided type 1 error of 5%. Hence, a sample size of 250 provided more than adequate power to detect a difference between the two populations. We estimated the mean and standard deviation of TSH levels in Tamale and Accra will be approximately 9.82 ± 1.64 and 4.18 ± 1.17, respectively [24]. Our secondary aim was to compare congenital hypothyroidism prevalence between infants in Accra and Tamale using TSH values as a measure of congenital hypothyroidism.

### Statistical analysis

Descriptive analyses were conducted on demographic and nutrition data. Categorical variables were described using proportions and most continuous measures were described using means and interquartile ranges (IQR). Maternal urine iodine concentrations and TSH levels were described using medians with 95% confidence intervals (CI) calculated by bootstrapping, as per the United Nations Children’s Fund (UNICEF) recommendations [25]. We used Chi-squared, t-tests or Wilcoxon rank tests as appropriate for comparisons. A Spearman correlation coefficient was employed to evaluate the correlation between maternal iodine levels and infant TSH.

We then assesed variables which we hypothesized would be associated with infant TSH using univariate linear regressions models. These variables included: maternal urine iodine concentration (log-transformed to account for its skewedness), maternal age, location (Accra or Tamale), mother’s educational status (dichotomized into none or some formal education, with some formal education defined as any amount of schooling from lower primary school (class one to class three) through to tertiary education), parity (primiparous/first-time or multiparous), infant age (in weeks), infant birthweight, infant sex, maternal use of iodized salt, maternal use of bouillon cube and maternal seafood intake. Variables significant at the *P* < 0.1 level in the univariate analysis were included in a multivariable regression model to identify the factors with independent effect on infant TSH. *P* < 0.05 was considered significant for the multivariable regression.

Further, we performed sensitivity analysis by excluding outliers of maternal urine iodine concentration and by dichotomizing maternal urine iodine concentration into high and low iodine levels, using the WHO cutoff of 100 μg/L. We also attempted to conduct sensitivity analysis of infant TSH by dichotomizing TSH into < 10 μIU/ml and ≥ 10 μIU/ml. We used TSH of 10 μIU/ml for this analysis because normal infant TSH in the first month of life is < 10 μIU/ml across many different populations [3]. However, only 13 out of 258 infants for whom TSH data was available fell above the cutoff of 10, thus further regression modeling on this small sample was not performed.

## Results

### Subject demographics

Two-hundred-fifty women and their infants were recruited, consisting of 125 mother-baby pairs from each site. No mother was excluded due to her medical history. After excluding mothers whose infants were younger than 3 days or older than 30 days, there were 123 women in Accra and 122 in Tamale for whom demographic data and urine samples were available. One mother and infant pair did not have demographic information available, yet, they provided maternal urine and infant bloodspot samples. Due to twin deliveries, there were 128 infants in Accra and 130 in Tamale for whom TSH data was available. Two infants, each one a part of a set of twins in Tamale died prior to the study. Their mothers provided demographic data on them, in addition to data on their live twin sibling, however, there was no TSH available for them.

Mean maternal age was 29.6 years (inter-quartile range (IQR) 25–34 years) in Accra and 29.3 years (IQR 25–33 years) in Tamale (Table 1). In Accra, 46% of participants were first-time mothers, compared to 30% in Tamale. Most participants in Accra were educated beyond primary school, with only 2% reporting no formal education. Women in Tamale, conversely, had a bi-modal distribution of their educational status, with 32% reporting no formal education and 33% reporting tertiary education.

**Table 1:** Maternal Demographics, Nutritional Information and Iodine Status by Location

Demographic	Accra *N* = 123 (%)	Tamale *N* = 122 (%)
Mother’s Age		
Years, [*mean, IQR*]	29.6 (25–34)	29.3 (25–33)
Educational Level		
None	3 (2.4)	39 (32.0)
Lower primary	4 (3.3)	3 (2.5)
Upper primary	5 (4.1)	3 (2.5)
Junior High School	46 (37.4)	17 (13.9)
Senior High School	36 (29.3)	20 (16.4)
Tertiary	29 (23.6)	40 (32.8)
Parity		
1st time	56 (45.0)	37 (30.0)
Multiparous	67 (55.0)	85 (70.0)
*Nutrition Information*		
Salt Type Used at Home		
Rock salt alone	12 (9.8)	22 (18.0)
Ground up salt alone	48 (39.0)	63 (51.6)
Rock and Ground up salt	63 (51.2)	37 (30.3)
Iodized Salt Use		
Yes	110 (89.4)	92 (75.4)
No	12 (9.8)	29 (23.8)
I don’t know	1 (0.8)	1 (0.8)
Frequency of Iodized Salt Use		
All the time	51 (46.4)	61 (66.3)
Most of the time	19 (17.3)	6 (6.5)
Sometimes	37 (33.6)	22 (23.9)
Rarely	3 (2.7)	3 (3.26)
Bouillon Cube Use		
Yes	110 (89.4)	114 (93.4)
No	13 (10.6)	8 (6.6)
Bouillon Cube Use Frequency		
All the time	33 (30.0)	79 (69.3)
Most of the time	8 (7.3)	8 (7.0)
Sometimes	66 (60.0)	26 (22.8)
Rarely	3 (2.7)	1 (0.9)
Sea Food Use		
Yes	117 (95.1)	117 (95.9)
No	6 (4.9)	5 (4.1)
Seafood Use Frequency		
Daily	23 (19.7)	53 (45.3)
Several times a week	16 (13.7)	50 (42.7)
Once or twice a week	55 (83.3)	11 (16.7)
Less than once/ week	23 (19.7)	3 (2.6)
*Iodine status*		
Maternal Urine Iodine Concentration		
μg/L, [*median, 95% CI*]	141.0 (115.7–166.5)	142.5 (125.1–160.0)
Iodine Status		
Severe iodine deficiency	1 (0.8)	0 (0)
Moderate iodine deficiency	9 (7.3)	8 (6.6)
Mild iodine deficiency	28 (22.8)	28 (23.1)
Iodine sufficient	49 (39.8)	54 (44.6)
Above requirements	24 (19.5)	15 (12.4)
Excessive iodine	12 (9.8)	16 (13.2)

Demographic information was available for 127 infants in Accra and 132 in Tamale (Table 2). There were similar proportions of female infants between the 2 sites (49 and 48% in in Accra and Tamale, respectively). The mean age (IQR) of infants was 12.3 (8–14) days in Accra and 12.2 (8.5–15) days in Tamale. Mean birthweight (IQR) of infants born in Accra was 3.1 (2.7–3.5) kg and 2.9 (2.5–3.2) kg for infants born in Tamale.

**Table 2:** Infant Demographics and TSH status by Location

Demographic	Accra *N* = 127	Tamale *N* = 132
Infant Sex		
Female	62 (48.8)	63 (47.7)
Male	65 (51.2)	69 (52.3)
Infant Age		
Days*, [mean, IQR]*	12.3 (8–14)	12.2 (8.5–15)
Infant Birth Weight		
Kg*, [mean, IQR]*	3.1 (2.7–3.5)	2.9 (2.5–3.2)
Thyroid Stimulating Hormone Level		
μIU/ml*, [median, 95% CI]*	4.7 (3.9–5.5)	3.5 (3.3–3.6)

*N for infant TSH in Tamale = 130, N for infant demographics in Tamale =132. This is because 2 babies, each a part of a twin set died prior to the study, hence no TSH data was available for them

### Nutritional knowledge and history

Approximately 98.4% of women in Accra reported having heard about iodized salt and 84.4% of women in Tamale said they had heard of iodized salt. Media, specifically television and radio were the most common sources of information about iodized salt. The proportion of women who reported using rock salt alone was almost twice in Tamale compared to Accra (18.0% vs. 9.8%, respectively). A greater proportion of women in Accra (89.4%) reported using iodized salt than in Tamale (75.4%). Of those who use iodized salt, 46.4% in Accra use it all the time vs. 66.3% in Tamale (Table 1).

Approximately 89.4% of women in Accra use bouillon cubes compared to 93.4% in Tamale, with women in Tamale being more consistent in using it all the time. Seafood intake was high across both locations - 95.1% of women in Accra and 95.9% in Tamale reported eating seafood. Women in Tamale, however, eat seafood more consistently - 45.3% of them reported eating seafood daily vs. 19.6% of women in Accra.

### Iodine status

The median (95% CI) urine iodine concentration for women in Accra was similar to those for women in Tamale: 141.0 (115.7–166.5) μg/L vs. 142.5 (125.1–160.0)) μg/L, respectively. The difference in medians was − 1.5 μIU/ml (95% CI: − 32.2 - 29.2) and was not statistically significant. Approximately 30.9% of women in Accra and 29.7% of women in Tamale were iodine deficient, with most of them falling into the “mild iodine deficiency” category. Approximately 29.3% of mothers in Accra and 25.7% in Tamale had urine iodine concentrations that were above the WHO recommended levels for nursing mothers.

### TSH status

The median TSH (95% CI) for the 127 infants born in Accra and included in the final analysis was 4.7 μIU/ml (3.9–5.5). In Tamale, the median TSH of the 130 infants was 3.5 μIU/ml (3.3–3.6). The difference in medians was 1.3 μIU/ml (95% CI: 0.5–2.1) and was statistically significant, *p* = 0.002. No infant had a TSH > 17 μIU/ml; thus, none of them met our criteria for congenital hypothyroidism (Table 2).

### Correlations

**Fig. 1:**
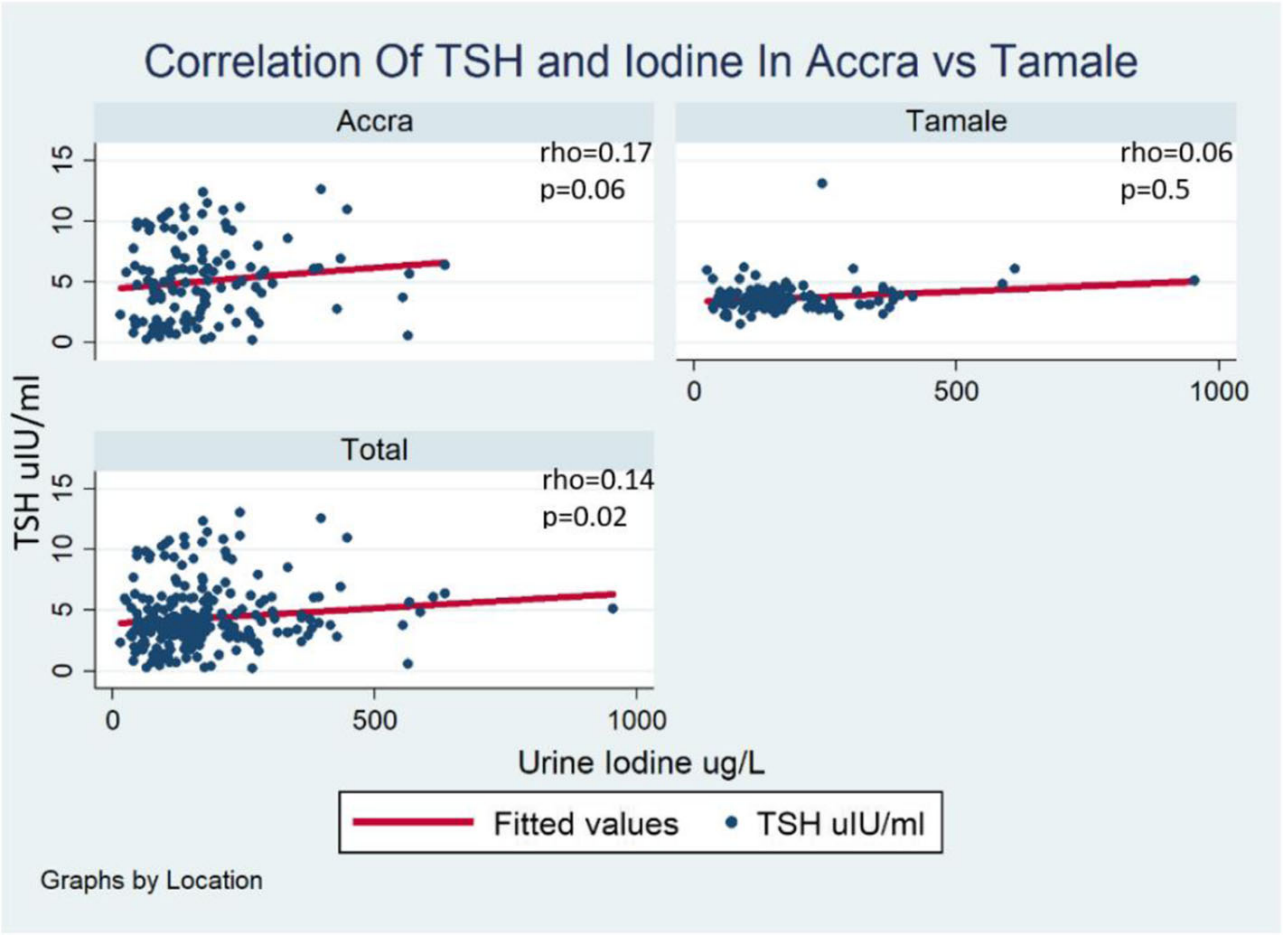
Correlation plot of infant TSH and maternal urine iodine concentration

Spearman correlation analysis showed a weakly positive correlation between infant TSH and maternal urine iodine concentration, with an overall correlation coefficient of 0.1, *p* = 0.02. When stratified by location, this weak positive correlation endured, with a correlation coefficient (rho) of 0.2, *p* = 0.06 in Accra and 0.06, *p* = 0.5 in Tamale (Fig. 1).

### Regression analyses

Univariate analyses of factors that may be associated with log-transformed maternal urine iodine concentration revealed that mothers with some formal education had higher iodine levels (*p* = 0.006). Mothers who used iodized salt tended to have higher iodine levels, though this relationship did not reach statistical significance (*p* = 0.06).

**Table 3:** Univariate and Multivariable Analysis of Factors Associated with Thyroid Stimulating Hormone

	Univariate Analysis			Multivariable Analysis		
	Coeff	95% CI	*p*-value	Coeff	95% CI	*p*-value
Log Iodine	0.48	[0.00,0.96]	0.048	0.45	[−0.02,0.92]	0.059
Mother’s Age	0.06	[0.00,0.11]	0.035	0.05	[− 0.00,0.10]	0.063
Living in Accra	1.36	[0.76,1.96]	< 0.001	1.26	[0.60,1.91]	< 0.001
Having Some Formal Education	1.06	[0.24,1.87]	0.011	0.18	[−0.71,1.06]	0.693
Multiparous Mother	−0.20	[−0.85,0.45]	0.543			
Infant’s Age in Weeks	0.31	[−0.07,0.69]	0.110			
Infant’s Birthweight	−0.16	[−0.72,0.40]	0.580			
Male Infant	0.36	[−0.26,0.98]	0.254			
Use of Iodized Salt	0.02	[−0.77,0.82]	0.959			
Use of Bouillon Cube	−0.98	[−2.09,0.13]	0.083			
Seafood Intake	−0.13	[−1.56,1.30]	0.858			

Univariate analyses of factors which may be associated with TSH levels revealed that infants who had older mothers (*p* = 0.035), mothers with some formal education (*p* = 0.011), mothers with higher iodine levels (*p* = 0.048) and who were born in Accra (*p* < 0.001) had higher TSH levels. Multivariable linear regression showed that living in Accra was the only factor that remained significantly associated with increasing infant TSH (*p* < 0.001) (Table 3).

**Fig. 2:**
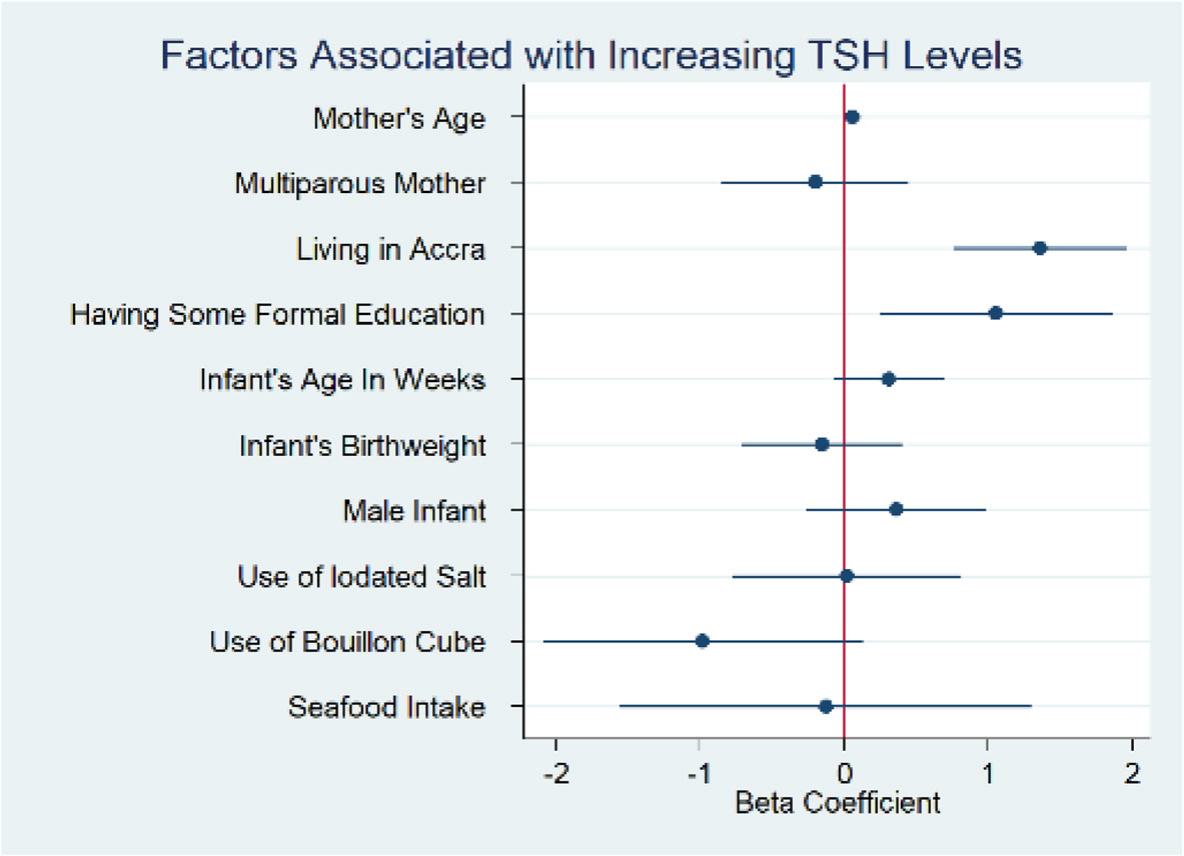
Coefficient Plot Univariate Analysis Factors Associated with Thyroid Stimulating Hormone (TSH)

**Fig. 3:**
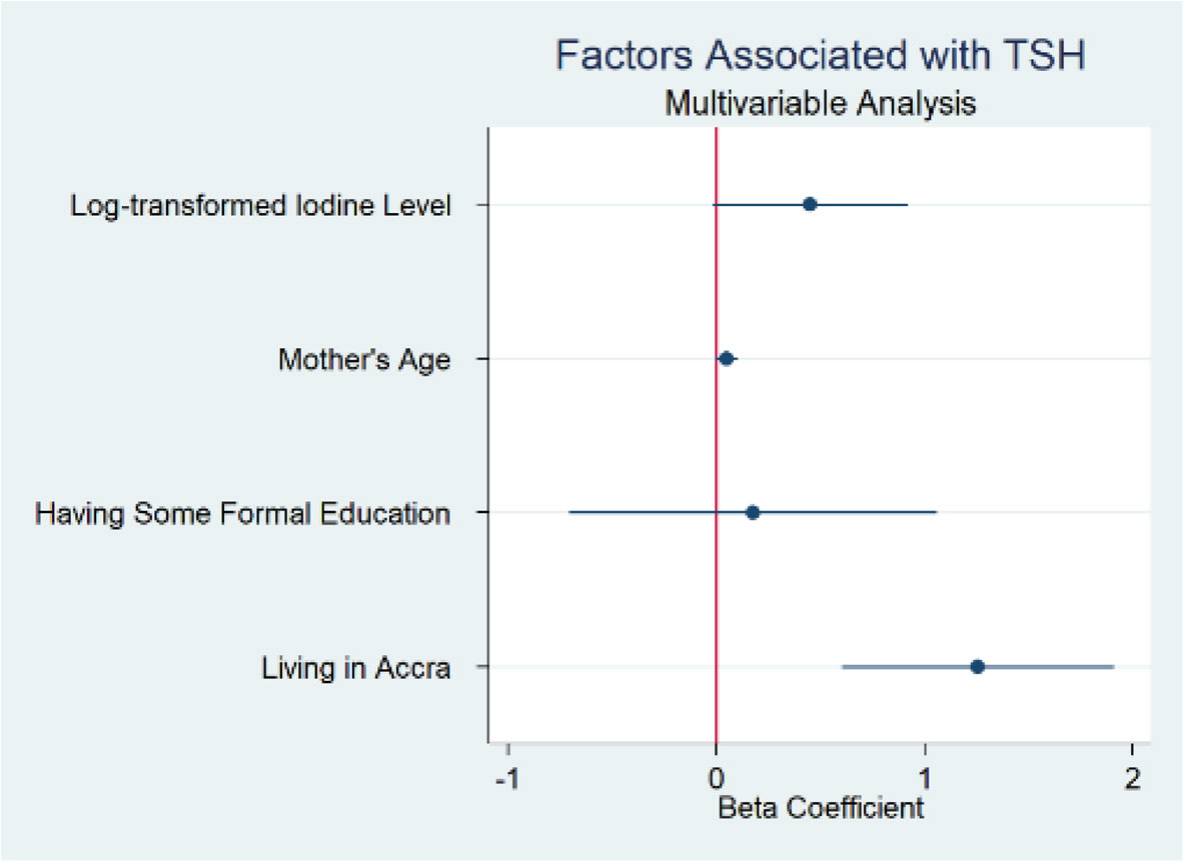
Coefficient Plot Multivariable Analysis Factors Associated with Thyroid Stimulating Hormone (TSH)

Sensitivity analyses were performed with maternal urine iodine concentrations dichotomized into high (urine iodine concentration ≥ 100 μg/L) vs. low iodine levels (urine iodine concentration < 100 μg/L). Findings were similar to the linear regression model. Mothers with any formal education had significantly higher urine iodine concentrations, and those who used iodized salt also trended toward having higher iodine levels (Figs. 2 and 3). Further sensitivity analyses excluding outliers of urine iodine concentration in each regression model did not change the outcomes.

## Discussion

In this study we found that intake of iodine from iodized salt, bouillon cube and seafood was generally high, though 30% of mothers in our population were iodine deficient. Some formal education among mothers was protective against iodine deficiency. Infant TSH was significantly different between the two sites, with a median TSH in Accra of 4.7 μIU/ml (95% CI: 3.9–5.5) and 3.5 μIU/ml (95% CI: 3.3–3.6) in Tamale and a difference in medians of 1.3 μIU/ml (95% CI: 0.5–2.1, *p* = 0.002). There was an unexpected positive correlation between maternal urine iodine and infant TSH.

There was no case of congenital hypothyroidism identified in this study. This is not surprising, given our small sample size of 258 infants and the reported incidence rate of congenital hypothyroidism of 1:2000–1:4000 [2]. Given this reported incidence of congenital hypothyroidism, a study powered to detect multiple cases of congenital hypothyroidism will require a much larger sample size than this study could provide. The secondary aim of this study was thus an exploratory aim. There is some debate in the literature over what cut-off should be selected for screening, with some researchers suggesting that infants with TSH between 15 and 20 μIU/ml or 17–20 μIU/ml may be missed cases of mild congenital hypothyroidism. Infants with true positive cases of congenital hypothyroidism often have TSH > 30 μIU/ml, though a small percentage of those with TSH values between 17 μIU/ml-30 μIU/ml will be false negatives [20]. However, in our cohort, only 13 out of 258 infants had TSH > 10 μIU/ml, and none reached a TSH of 15 μIU/ml. This evaluation makes it unlikely that a true case of congenital hypothyroidism was missed in this study.

Much of the available data regarding iodine deficiency in Ghana comes from small studies like ours which report some percentage of iodine deficiency in their populations [14, 26, 27]. This is in contrast to a 2015 national iodine survey in Ghana which reported median urine iodine concentration in women in fertile age was generally at or above sufficient levels, though there were differences based on location [28]. Overall, the high intake of iodine-containing foods in our population is reassuring and suggests that prior public health efforts to encourage the use of iodized salt have been generally successful. Further, bouillon cubes, which are commonly used in Ghana, represent a major source of iodine in the diet [29].

Nevertheless, 30% of our subjects were iodine deficient, which raises concern. The discrepancy between our 30% prevalence of iodine deficiency and the national data suggesting sufficient or above sufficient iodine status may be due to the inherent problems with urine iodine concentrations in individuals and small populations. Still, the fact that not all of our participants reported consumption of iodine containing products, and those who did were not always consistent, suggests that there remains a real risk of iodine deficiency in individuals and in some populations. Consumers in Ghana have easy access to rock salt as well as iodized salt. Rock salt in Ghana is typically mined from the sea or ground and is not fortified with iodine. Use of this unfortified rock salt is not ideal for the population, yet it remains an option.

Some formal education among mothers was protective against iodine deficiency. This finding may be because educated mothers are more likely to read or seek out information. Education may also be a confounder in this population, and may represent a marker of socio-economic status and access to certain foods.

There was a weakly positive correlation between maternal urine iodine and infant TSH. This was unexpected and goes against prevailing scientific knowledge, which suggests that as pregnant mothers have more iodine in their diets, their infants should have lower TSH, as increasing TSH is a marker of worsening thyroid function. However, other studies, at least one in Denmark and another in Australia also found unexpectedly higher TSH in infants born to mothers with higher iodine levels or mothers who had iodine supplementation. Some researchers suggest that infants born in areas with overall mild iodine deficiency may be more susceptible to iodine’s inhibitory effects and have a higher but clinically non-significant TSH level [30, 31].

There are several factors which muddy this picture, including our small sample size, the fact that the TSH of infants change very quickly and many centers have age-specific norms for their population. Ghana does not have any age-specific norms for TSH in infants, so it is difficult to know if these infants’ TSH values are representative of the general population. Further, as mentioned above, a mother’s iodine on the day of the study may not accurately reflect her iodine status during her pregnancy.

This study was a multi-site study within larger populations which have some general differences. Accra is urban, is the capital city of Ghana, and is along the Atlantic coast. Tamale is a smaller city surrounded by more rural surroundings, is not along the coast and is in the Northern region which traditionally has had more iodine deficiency [15]. This fact that our two populations came from such different areas is a strength of our study. Furthermore, as mentioned above, there is sparse available data on thyroid function of neonates in Ghana and there is no available data on the relationship between mother’s iodine status and neonatal thyroid function of infants in this population. This study, though small, is novel and probes an important but understudied area of literature. It serves as a feasibility study which shows that TSH screening can be done in the Ghanaian context, though much work will need to be done to get it going. Finally, though this study did not directly measure attitudes towards potential newborn screening, the process was well received by parents and mothers were engaged in the study. This represents an encouraging and important first step to newborn screening for congenital hypothyroidism in Ghana.

Limitations of our study include the small sample size and the cross-sectional design, which limits causal attribution. In addition, urine iodine, while the standard means of testing iodine status, is much more suited to large, population-wide studies, and this may impact the validity of our findings.

## Conclusion

In conclusion, the median and mean TSH of infants in Accra was higher than that of infants in Tamale. About 30% of mothers in both locations were iodine deficient, though the intake of iodine-containing foods was high. Maternal education appeared to be protective against iodine deficiency in this population, and there was an unexpected weakly positive correlation between maternal urine iodine and infant TSH. Larger longitudinal studies are needed to establish age-specific TSH norms in infants, to better establish the iodine status of the population, and further delineate the relationship between maternal iodine status and infant thyroid function. There are multiple factors which bode well for newborn screening of infants in Ghana, such as increasing interest from physicians and parents, and these represent opportunities for further work towards newborn screening.

## Data Availability

The datasets used and/or analyzed during the current study are available from the corresponding author on reasonable request.

## Abbreviations

CDC EQUIP: Centers for disease control and prevention ensuring the quality of urinary iodine procedures
CI: Confidence interval
IQR: Interquartile range
KBTH: Korle-Bu teaching hospital
NBS: Newborn screen
RedCAP: Research electronic data capture
T4: Thyroxine
TSH: Thyroid stimulating hormone
TTH: Tamale teaching hospital
UNICEF: United Nations children’s fund
WHO: World health organization
